# Mild behavioral impairment: measurement and clinical correlates of a novel marker of preclinical Alzheimer’s disease

**DOI:** 10.1186/s13195-021-00949-7

**Published:** 2022-01-05

**Authors:** Byron Creese, Zahinoor Ismail

**Affiliations:** 1 Medical School, College of Medicine and Health, University of Exeter, Exeter, UK; 2 Departments of Psychiatry, Clinical Neurosciences, Community Health Sciences, and Pathology, Hotchkiss Brain Institute, University of Calgary, Calgary, Alberta Canada

**Keywords:** Mild behavioral impairment, Neuropsychiatric symptoms, Preclinical AD, Cognition, Biomarkers

## Abstract

**Background:**

Late-life onset neuropsychiatric symptoms are established risk factors for dementia. The mild behavioral impairment (MBI) diagnostic framework was designed to standardize assessment to determine dementia risk better. In this Mini Review, we summarize the emerging clinical and biomarker evidence, which suggests that for some, MBI is a marker of preclinical Alzheimer’s disease.

**Main:**

MBI is generally more common in those with greater cognitive impairment. In community and clinical samples, frequency is around 10–15%. Mounting evidence in cognitively normal samples links MBI symptoms with known AD biomarkers for amyloid, tau, and neurodegeneration, as well as AD risk genes. Clinical studies have found detectable differences in cognition associated with MBI in cognitively unimpaired people.

**Conclusion:**

The emerging evidence from biomarker and clinical studies suggests MBI can be an early manifestation of underlying neurodegenerative disease. Future research must now further validate MBI to improve identification of those at the very earliest stages of disease.

## Background

There exists an urgent need to improve early detection of Alzheimer’s disease (AD) in order to optimize interventions in preclinical phases and potentially improve the success rates of trials of disease-modifying therapies. Access to biomarkers for preclinical disease detection is improving, but screening of cognitively asymptomatic populations remains inefficient and costly. Therefore, attention has turned to enrichment strategies to identify those most likely to screen positive; these should be low-cost, quick, and easy to administer at large. Screening on late-life onset neuropsychiatric symptoms (NPS) may represent such a strategy.

Later-life NPS are reliably associated with a greater risk of mild cognitive impairment (MCI) and dementia [1–8]. This evidence motivated the development of the mild behavioral impairment (MBI) diagnostic framework, which operationalizes and standardizes the assessment of NPS in older adults, to improve risk determination of all-cause dementia. It is well appreciated that emergent behavioral symptoms can presage frontotemporal dementia. However, the behavioral prodrome is less appreciated in AD, and thus, AD is the focus of this review. Here, we introduce MBI and its measurement and review major clinical and biomarker research, which suggests that MBI can represent an early manifestation of AD.

### Main

MBI is a later-life onset neurobehavioral syndrome that may emerge at any point along the pre-dementia spectrum, from normal cognition to subjective cognitive decline (SCD) through to MCI. MBI represents a neurobehavioral axis, alongside the traditional neurocognitive axis, of pre-dementia symptoms. MBI is not a competing construct to MCI, but rather, a complementary behavioral analog. Just as emergent cognitive symptoms in late life can reflect neurodegeneration, so can emergent behaviors. These behaviors in MBI are clustered into five domains (Table 1).

**Table 1:** ISTAART research diagnostic criteria for Mild Behavioral Impairment

1. Changes in behavior or personality observed by patient, informant, or clinician, starting later in life (age ≥ 50 years) and persisting at least intermittently for ≥6 months. These represent a clear change from the person’s usual behavior or personality as evidenced by at least one of the following:	
a. Decreased motivation (e.g., apathy, aspontaneity, indifference)	
b. Affective dysregulation (e.g., anxiety, dysphoria, changeability, euphoria, irritability)	
c. Impulse dyscontrol (e.g., agitation, disinhibition, gambling, obsessiveness, behavioral perseveration, stimulus bind)	
d. Social inappropriateness (e.g., lack of empathy, loss of insight, loss of social graces or tact, rigidity, exaggeration of previous personality traits)	
e. Abnormal perception or thought content (e.g., delusions, hallucinations)	
2. Behaviors are of sufficient severity to produce at least minimal impairment in at least one of the following areas:	
a. Interpersonal relationships	
b. Other aspects of social functioning	
c. Ability to perform in the workplace	
The patient should generally maintain his/her independence of function in daily life, with minimal aids or assistance.	
1. Although comorbid conditions may be present, the behavioral or personality changes are not attributable to another current psychiatric disorder (e.g., generalized anxiety disorder, major depression, manic or psychotic disorders), traumatic or general medical causes, or the physiological effects of a substance or medication.	
2. The patient does not meet criteria for a dementia syndrome (e.g., Alzheimer’s disease, frontotemporal dementia, dementia with Lewy bodies, vascular dementia, other dementia). MCI can be concurrently diagnosed with MBI.	

Abbreviations: ISTAART (Alzheimer's Association International Society to Advance Alzheimer's Research and Treatment)

In 2016, an Alzheimer’s Association working group published diagnostic criteria for MBI (Table 1), and the Mild Behavioral Impairment Checklist (MBI-C) was developed to capture the full symptom spectrum in accordance with the criteria [9, 10]. The MBI-C is free, has been validated in clinical and community samples, and can be administered in person, by telephone, or unsupervised online [11–16]. Two primary questions relating to assessment using the MBI-C remain outstanding, specifically the cut points for case ascertainment, and the use of self- versus informant-reports. Regarding MBI-C cut points, 6.5 and 8.5 in MCI and SCD samples show good sensitivity and specificity for gold-standard clinical diagnosis of MBI [12, 13]. However, there is no similar research for individual MBI domains. Most studies use informant-reports but self-reported MBI is also associated with worse cognition, highlighting its potential importance [17]. Although symptoms are reported at a similar frequency when comparing self- and informant-reports, the two are only weakly correlated, and therefore possibly capture different groups [11]. The role of anosognosia has not been explored fully in MBI, an understanding of which may provide further insights into the sources of information, and help direct the appropriate use self- or informant-reports [18].

Prevalence estimates of MBI vary considerably according to the instrument used and the sample studied. Generally, MBI is more prevalent with greater cognitive impairment. Studies using the Neuropsychiatric Inventory (NPI, developed for established dementia), place prevalence as high as 49–85% in MCI and 28–75% in pre-MCI samples [19, 20]. Studies using the MBI-C yield more conservative estimates of 14% in MCI and 6–9% in pre-MCI [12, 13, 16, 17]. These estimates are more accurate due to MBI-C stipulations that symptoms emerge de novo in later life and persist for at least 6 months. These requirements result in better specificity for symptoms representing the manifestations of neurodegenerative disease, with transient symptoms and those representing life stressors less likely to be included [21]. Studies using the MBI-C in memory clinics report prevalence of 28–37% in SCD and 47–54% in MCI [15, 22], emphasizing the clinical significance of behavioral symptoms [23]. Impulse dyscontrol and affective dysregulation have been reliably identified as the two most common individual MBI domains by multiple independent studies [11, 13, 19, 20, 22, 24–26].

Longitudinal and cross-sectional studies have consistently linked MBI with poorer cognition and progression to MCI and dementia [16, 17, 21, 25, 27–30]. Studies of detailed neuropsychology in cognitively normal (CN) individuals have shown differences in executive function, attention, working memory, and episodic memory associated with MBI across a number of independent studies [16, 17, 25, 30]. Interestingly, one study has shown MBI with MCI to confer a greater degree of episodic memory impairment than MCI without MBI but it is not known to what extent these broad early cognitive deficits might indicate risk for specific types of neurodegenerative disease [25]. Effect sizes in these studies were small-to-medium but the findings are important and suggest detectable differences in cognition exist. Longitudinal studies are required to determine whether MBI emerges in advance or after these subtle changes.

In a sample of over 4000 people stratified by MBI status, AD genetic risk was associated with cognitive performance in those with MBI but not in those without. These findings suggest screening on MBI may enrich samples for a cognitive phenotype associated with AD biomarkers [31]. While encouraging, no firm conclusions can be drawn about the etiology underlying genetic and cognitive associations. However, emerging biomarker evidence provides more concrete links with AD neuropathology.

A nascent literature operationalizing MBI using the NPI has linked symptoms with plasma neurofilament light, suggesting accelerated neurodegeneration, and has also shown a modest association with plasma Aβ_42_/Aβ_40_ [32, 33]. There is also early evidence that impulse dyscontrol is associated with atrophy in the entorhinal cortex [34], a finding also reported in a different sample focused on a small number of brain regions selected a priori [35]. Greater medial temporal lobe atrophy was also associated with total MBI-C score in a subsequent study; however, these findings should be considered exploratory as only uncorrected *p*-values were presented in the small sample of 34 participants [36]. Amyloid PET is a gold standard biomarker of AD and in a recent landmark study of CN older adults, MBI score was correlated with both global and striatal Aβ burden [24]. Moreover, of the 7 participants scoring above 8 on the MBI-C, 5 (71%) were amyloid positive, compared with 25% of those scoring < 8 [24]. There was no association with tau PET in this sample. A separate study of Aβ positive CN participants (i.e., preclinical AD), found higher MBI-C scores were associated with tau PET uptake in the entorhinal cortex and hippocampus, as well as higher CSF p-tau_181_ [37]. Reconciling these seemingly contradictory studies requires an appreciation of the difference in amyloid positivity. Given that amyloid is likely required for abnormal tau, the sampling difference with respect to amyloid positivity in the two studies may explain the discrepancy. Collectively, then, evidence from MRI and PET implicates medial temporal lobe structures. Interestingly, tau PET was not associated with delayed recall in the preclinical AD study, suggesting the MBI association did not simply reflect memory deficits. One explanation for the association is the “agitation network” proposed by Rosenberg et al. that comprises medial temporal lobe structures (including the hippocampus and amygdala) and frontal and anterior cingulate cortices [38]. Alternatively, given that the sample had preclinical AD, and not prodromal AD/MCI, by definition cognition should be normal, with the behavioral changes representing sequelae of amyloid and/or tau. Further studies are needed to explore these findings.

These initial biomarker findings are exciting, but it is important to highlight that the literature remains in its infancy. Specifically, small sample sizes and only a small number of independent studies remain a limitation. Functional imaging studies are also needed to elucidate specific brain networks correlated with MBI symptoms. Notwithstanding these limitations, the literature is promising, and these data provide a clear rationale for empirical validation of MBI as a sample enrichment strategy in longitudinal studies using established and novel biomarkers [39, 40]. Furthermore, the exploration of associations with non-AD biomarkers is essential given that depending on the nature of the clinical sample, some studies have shown a substantial proportion of patients with MBI can progress to Lewy body and frontotemporal dementia as well [29, 30, 41].

## Conclusions

An urgent need exists for a clinical marker to help identify people in the early stages of AD, alongside biomarker screening. MBI criteria provide a standardized approach to NPS assessment and meet the basic requirements of an enrichment strategy; assessment is quick, cheap, and scalable to large samples. Research is needed to optimize measurement, focus on individual MBI domains, and reconcile self- and informant-reports. Nonetheless, mounting evidence suggests that MBI is associated with cognitive decline and a variety of AD biomarkers in cognitively normal samples, positioning it as a novel marker of preclinical disease. Future research must now understand MBI associations with accumulating AD pathology to validate it as a prognostic enrichment strategy.

## Data Availability

Not applicable.

## Abbreviations

AD: Alzheimer’s disease
Aβ: Amyloid beta
CSF: Cerebrospinal fluid
MBI: Mild behavioral impairment
MBI-C: Mild Behavioral Impairment Checklist
MCI: Mild cognitive iImpairment
NPS: Neuropsychiatric symptoms
PET: Positron emission tomography
SCD: Subjective cognitive decline
